# Reduced chronic restraint stress in mice overexpressing hyperactive proteasomes in the forebrain

**DOI:** 10.1186/s13041-020-0548-y

**Published:** 2020-01-13

**Authors:** Ji Hyeon Kim, Ahbin Kim, Yejin Yun, Seoyoung Park, Jung Hoon Lee, Yong-Seok Lee, Min Jae Lee

**Affiliations:** 1 Department of Biomedical Sciences, Seoul National University Graduate School, Seoul, 03080 Korea; 2 Department of Biochemistry and Molecular Biology, Seoul National University College of Medicine, Seoul, 03080 Korea; 3 Department of Physiology, Seoul National University College of Medicine, Seoul, 03080 Korea; 4 Neuroscience Research Institute, Seoul National University College of Medicine, Seoul, 03080 Korea

**Keywords:** Proteasome, Gate, Chronic stress, Depression-like behavior, Oxidative stress

## Abstract

While chronic restraint stress (CRS) results in depression-like behaviors possibly through oxidative stress in the brain, its molecular etiology and the development of therapeutic strategies remain elusive. Since oxidized proteins can be targeted by the ubiquitin-proteasome system, we investigated whether increased proteasome activity might affect the stress response in mice. Transgenic mice, expressing the N-terminally deleted version of α3 subunit (α3ΔN) of the proteasome, which has been shown to generate open-gated mutant proteasomes, in the forebrain were viable and fertile, but showed higher proteasome activity. After being challenged with CRS for 14 d, the mutant mice with hyperactive proteasomes showed significantly less immobility time in the forced swimming test compared with their wild-type littermates, suggesting that the α3ΔN transgenic mice are resistant to CRS. The accumulation of ER stress markers, such as polyubiquitin conjugates and phospho-IRE1α, was also significantly delayed in the hippocampus of the mutants. Notably, α3ΔN mice exhibited little deficits in other behavioral tasks, suggesting that stress resilience is likely due to the degradation of misfolded proteins by the open-gated proteasomes. These data strongly indicate that not only is the proteasome a critical modulator of stress response in vivo but also a possible therapeutic target for reducing chronic stress.

## Main text

Depression is widely accepted to be closely linked with long-term stress, which stimulates the hypothalamic-pituitary-adrenal axis, thereby upregulating cortisol production in humans [1]. Chronic restraint stress (CRS) not only elevates blood corticosterone but also recapitulates the persisting depression-like behaviors in rodents [2]. CRS-induced mice showed increased reactive oxygen species generation and the resultant lipid peroxidation and protein carbonylation in the hippocampus [3]. This is consistent with the conclusions of a recently conducted meta-analysis—lower antioxidant and higher free radical and oxidatively damaged protein levels in the serum of depressed patients [4]. However, the etiological mechanism coupling oxidative stress to depression is largely undetermined.

The 26S proteasome comprises the 28-subunit 20S and the 19-subunit 19S particles. At the interface, between the 20S and 19S of the substrate translocation channel, the N-terminal tails of the α subunits form the gate, blocking substrate access into the proteolytic chamber of the 20S [5]. Our previous studies, using mammalian cells, have shown that deletion of the N-terminal tail of the α3 subunit (α3ΔN) resulted in the conformational destabilization of the gate and facilitated substrate entry into the interior active sites [6]. We found that these open-gated mutant proteasomes, both 20S and 26S, were indeed hyperactive in substrate hydrolysis and showed accelerated degradation of oxidized proteins in the cell [6]. Proteasome-activating small molecules and RNA aptamers have been shown to exhibit cytoprotective effects against oxidative stress [7, 8].

Oxidized proteins form a class of intrinsically disordered proteins, which can be degraded by the 20S proteasome without ubiquitination. In addition, exposure to oxidative stress results in global ubiquitination of oxidized proteins with Lys48-linked polyubiquitin (polyUb) chains and their subsequent degradation by the 26S proteasome [9]. Notably, non-degradable Lys63-polyUb chains also appeared to have a regulatory role during the early phase of the oxidative stress response [10]. These results suggest that proteasome activity appears to be closely related with cellular resistance to oxidative stress [11]. Consistent with these results, mild inhibition of proteasome activity was shown to result in depression-like symptoms in mice [12].

**Fig. 1:**
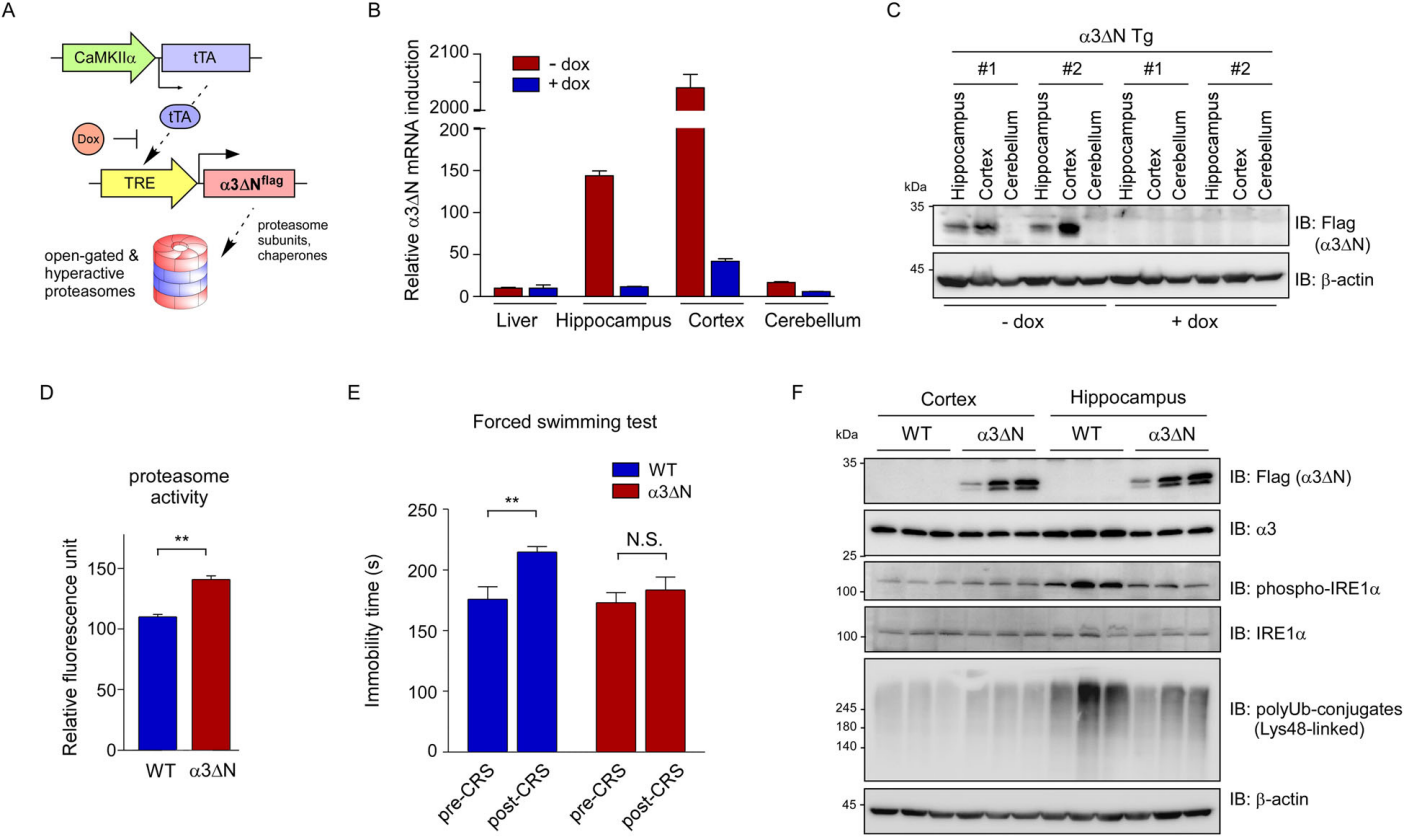
Enhancing proteasome activity in the brain may lessen chronic restraint stress (CRS). **a** A genetic scheme of neuron-specific expression of open-gated proteasomes. A flag-tagged form of α3 mutant, with a deleted N-terminal 9-residue tail (α3ΔN), was overexpressed by CaMKIIα promoter-driven transcriptional activator (tTA), in the absence of doxycycline (dox). **b** mRNA levels of α3ΔN from CaMKIIα-tTA/TRE-α3ΔN double transgenic (α3ΔN Tg) mice were measured with quantitative RT-PCR. Different brain regions were analyzed, which revealed little expression of α3ΔN in the cerebellum. Liver tissue was used as a negative control. Transgene expression was virtually completely abrogated with the administration of dox in drinking water. β-Actin was used as the loading control. **c** As in (**b**), except that α3ΔN proteins were analyzed with SDS-PAGE/immunoblotting (IB), using whole cell lysates from the indicated regions of the brain. The level of α3ΔN^flag^ protein was largely proportional to that of α3ΔN mRNA. **d** Proteasome activity in whole brain extracts, from wild-type (WT) and α3ΔN Tg mice, was measured using the fluorogenic suc-LLVY-AMC reporter substrate (mean ± SD from three independent experiments). **, *p* < 0.01 (two-tailed Student’s *t* test). **e** Immobility times in the forced swimming test before CRS and post-CRS day 14 (2 h per day). *N* = 5 for WT, *N* = 9 mice for Tg. **, *p* < 0.01 (two-tailed Student’s *t* test) N.S., not significant. **f** Less accumulation of polyubiquitin conjugates and phosphorylated IRE1α in the hippocampus of α3ΔN Tg mice than in WT controls. Cortex and hippocampus were isolated after 14 d of CRS and subjected to SDS-PAGE/IB analysis, using the indicated antibodies

To investigate whether the proteasome is involved in CRS-mediated biological effects in vivo and elevation of proteasome activity could delay the pathological progression of depression-like behaviors, we first generated transgenic mice expressing α3ΔN with a flag tag under the control of the TRE promoter and then crossed this line with tTA-expressing transgenic mice driven by the forebrain-specific CaMKIIα promoter (Fig. 1a). We first measured the mRNA levels in the brain of the double transgenic (α3ΔN Tg) mice and detected 204- and 14.4-fold increase in the cortex and the hippocampus, respectively, compared to the level detected in the liver (Fig. 1b). Transgene expression in the α3ΔN Tg mice was effectively suppressed when 2 mg/mL doxycycline (dox) was added to the drinking water for 4 weeks (Fig. 1b). Transgene expression was kept at a low level in the cerebellum regardless of dox treatment (Fig. 1b). Correspondingly, immunoblotting analysis of whole tissue lysates showed that the level of α3ΔN^flag^ protein was higher in the cortex than in the hippocampus, and virtually undetectable in the cerebellum (Fig. 1c).

We have previously shown that mammalian cells stably overexpressing α3ΔN had enhanced proteasome activity, demonstrating that this originated from 20S gate opening rather than secondary allosteric modulation [6]. We assessed overall proteasomal activity in the brain using a fluorogenic substrate suc-LLVY-AMC [13], and found that proteasome activity was significantly elevated in the α3ΔN Tg mice (~ 1.32 times higher than wild-type, WT) (Fig. 1d). Although we did not confirm the overall integrity of the 26S proteasome, our data, including high α3ΔN expression and proteasome activity in mutant mice, suggested that the transgenic mice had functional open-gated mutant proteasomes in the brain.

To examine whether enhanced proteasome activity affects stress-related behaviors, α3ΔN Tg mice were challenged with restraint stress 2 h daily for 14 d, and were forced to swim for 6 min at day 16 [14]. The time of immobility, occurring during the last 5 min, was measured blind. WT and Tg mice showed comparable immobility time before CRS, suggesting that transgene expression does not affect the baseline depression-like behavior (Fig. 1e). After CRS, immobility time was significantly extended in WT mice as anticipated [15] (pre-CRS, 176.0 ± 10.3 s; post-CRS, 214.9 ± 4.6 s; unpaired t-test, *p* = 0.0085; *N* = 5; Fig. 1e). In contrast, α3ΔN mice, with hyperactive proteasomes in the forebrain, showed only comparable immobility time after undergoing CRS (pre-CRS, 173.0 ± 8.5 s; post-CRS, 183.6 ± 10.6 s; unpaired t-test, *p* = 0.4481; *N* = 9; Fig. 1e). This resilience to CRS in Tg mice resembles the effects of diverse antidepressants, in the forced swimming test [16].

Given these findings, we examined the changes in ubiquitin conjugates during chronic stress in wild-type and α3ΔN Tg mice. In the cortex, CRS had little or no effect on the levels of Lys48-linked polyUb species (Fig. 1f), which are tagged to oxidized proteins for proteasomal degradation [9]. In sharp contrast, CRS treatment induced a significant upregulation of prominent ER stress markers, including polyubiquitin species and phosphorylated IRE1α, in the hippocampus of wild-type mice ([17] and references therein). In addition, the accumulation of these proteins, which possibly reflects the consequence of oxidative stress during CRS, was dramatically reduced in the hippocampus of α3ΔN mice (Fig. 1f). We did not observe any significant change in proteasome level in the mice forebrains, before or after CRS.Considering the possibility that the observed resilience of α3ΔN mice against CRS was due to other behavioral abnormalities, such as alterations in anxiety level or general mobility, we performed a battery of behavioral tests which revealed that elevating proteasome activity does not affect locomotor activity, anxiety level, learning and memory in mice (Additional file 1: Figure S1).

Taken together, the present study indicates that proteasome activity in the hippocampus is a direct regulator of chronic stress response and that enhanced proteasome activity is beneficial for relieving chronic stress-induced oxidative stress in mice. Although the molecular etiology of chronic stress and the related adaptive signaling pathway need to be further elucidated, the mechanistic clues identified in this study may provide a new therapeutic strategy for the treatment of depression or chronic stress.

## Supplementary information

**Additional file 1: Figure S1.** Enhancing proteasome activity does not affect locomotive activity, basal anxiety and fear memory. **(A-B)** Mice were tested in open field (33 × 33 cm) for 20 min under dim light. **(A)** Distance moved measured across the 20-min test session in open field test. WT, *n* = 5 mice; α3ΔN Tg, *n* = 9 mice. Unpaired t-test, *p* = 0.2210. **(B)** Time spent in center (15 cm) zone in open field test. WT, n = 5 mice; α3ΔN Tg, n = 9 mice. Unpaired t-test, *p* = 0.5440. **(C)** Time spent in closed and open arm in elevated plus maze test. WT, n = 5 mice; α3ΔN Tg, n = 9 mice. Two-way ANOVA, effect of genotype, F_1, 12_ = 0.1428, *p* = 0.7122. **(D-E)** Mice were trained with three tone (2.8 kHz, 85 dB, 30 s)-shock (0.5 mA, 2 s co-terminated with the tone) pairings in fear conditioning apparatus. **(D)** Contextual fear memory was tested in the same training apparatus for 5 min at 24 h after the training. WT, *n* = 11 mice; α3ΔN Tg, n = 11 mice. Unpaired t-test, *p* = 0.4354. **(E)** Auditory fear memory was tested in another chamber with the same tone for 3 min at 48 h after the training. WT, n = 11 mice; α3ΔN Tg, n = 11 mice. Unpaired t-test, *p* = 0.2260. Only male mice were used for open field and elevated plus maze, whereas both male and female mice were used for fear conditioning tests. Data are mean ± SEM.

## Data Availability

All data generated or analyzed during this study are included in this published article.

## Abbreviations

CaMKIIα: calcium/calmodulin-dependent protein kinase type IIα
CRS: Chronic restraint stress
Dox: Doxycyclin
ER: Endoplasmic reticulum
IRE1α: Inositol-requiring enzyme 1α
polyUb: polyubiquitin
TRE: Tetracyclin response element
tTA: tetracyclin transactivator

## References

[1] Aguilera G (2011). HPA axis responsiveness to stress: implications for healthy aging. Exp Gerontol.

[2] Son H, Yang JH, Kim HJ, Lee DK (2019). A chronic immobilization stress protocol for inducing depression-like behavior in mice. J Vis Exp.

[3] Seo JS, Park JY, Choi J, Kim TK, Shin JH, Lee JK, Han PL (2012). NADPH oxidase mediates depressive behavior induced by chronic stress in mice. J Neurosci.

[4] Liu T, Zhong S, Liao X, Chen J, He T, Lai S, Jia Y (2015). A meta-analysis of oxidative stress markers in depression. PLoS One.

[5] Finley D, Chen X, Walters KJ (2016). Gates, channels, and switches: elements of the proteasome machine. Trends Biochem Sci.

[6] Choi WH, de Poot SA, Lee JH, Kim JH, Han DH, Kim YK, Finley D, Lee MJ (2016). Open-gate mutants of the mammalian proteasome show enhanced ubiquitin-conjugate degradation. Nat Commun.

[7] Lee JH, Shin SK, Jiang Y, Choi WH, Hong C, Kim DE, Lee MJ (2015). Facilitated tau degradation by USP14 Aptamers via enhanced proteasome activity. Sci Rep.

[8] Kim E, Park S, Lee JH, Mun JY, Choi WH, Yun Y, Lee J, Kim JH, Kang MJ, Lee MJ (2018). Dual function of USP14 Deubiquitinase in cellular proteasomal activity and Autophagic flux. Cell Rep.

[9] Manohar S, Jacob S, Wang J, Wiechecki KA, Koh HWL, Simoes V, Choi H, Vogel C, Silva GM (2019). Polyubiquitin chains linked by lysine residue 48 (K48) selectively target oxidized proteins in vivo. Antioxid Redox Signal.

[10] Silva GM, Finley D, Vogel C (2015). K63 polyubiquitination is a new modulator of the oxidative stress response. Nat Struct Mol Biol.

[11] Raynes R, Pomatto LC, Davies KJ (2016). Degradation of oxidized proteins by the proteasome: distinguishing between the 20S, 26S, and immunoproteasome proteolytic pathways. Mol Asp Med.

[12] Romero-Granados R, Fontan-Lozano A, Aguilar-Montilla FJ, Carrion AM (2011). Postnatal proteasome inhibition induces neurodegeneration and cognitive deficiencies in adult mice: a new model of neurodevelopment syndrome. PLoS One.

[13] Shin SK, Kim JH, Lee JH, Son YH, Lee MW, Kim HJ, Noh SA, Kim KP, Kim IG, Lee MJ (2017). Docosahexaenoic acid-mediated protein aggregates may reduce proteasome activity and delay myotube degradation during muscle atrophy in vitro. Exp Mol Med.

[14] Shoji H, Miyakawa T (2019). Increased depression-related behavior during the postpartum period in inbred BALB/c and C57BL/6 strains. Mol Brain.

[15] Chiba S, Numakawa T, Ninomiya M, Richards MC, Wakabayashi C, Kunugi H (2012). Chronic restraint stress causes anxiety- and depression-like behaviors, downregulates glucocorticoid receptor expression, and attenuates glutamate release induced by brain-derived neurotrophic factor in the prefrontal cortex. Prog Neuro-Psychopharmacol Biol Psychiatry.

[16] Gould TD (2009). Mood and anxiety related phenotypes in mice : characterization using behavioral tests.

[17] Ii Timberlake M, Dwivedi Y (2019). Linking unfolded protein response to inflammation and depression: potential pathologic and therapeutic implications. Mol Psychiatry.

