# Supplementary figures and images for: Reduced chronic restraint stress in mice overexpressing hyperactive proteasomes in the forebrain
Source: Mol Brain. 2020 Jan 13;13:4. doi: 10.1186/s13041-020-0548-y (PMC6958796; doi:10.1186/s13041-020-0548-y)

A

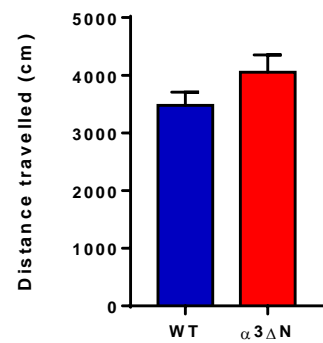

B

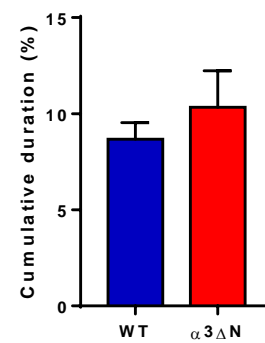

C

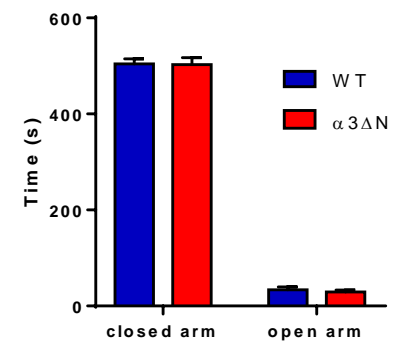

D

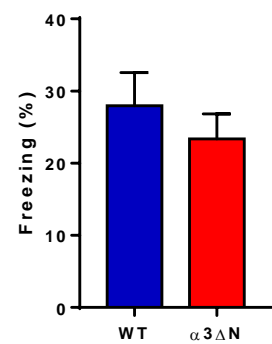

E

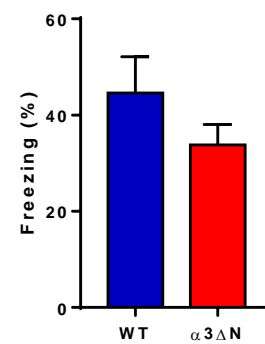

Supplement: Supplementary file 1 — Additional file 1: Figure S1. Enhancing proteasome activity does not affect locomotive activity, basal anxiety and fear memory. (A-B) Mice were tested in open field (33 × 33 cm) for 20 min under dim light. (A) Distance moved measured across the 20-min test session in open field test. WT, n = 5 mice; α3ΔN Tg, n = 9 mice. Unpaired t-test, p = 0.2210. (B) Time spent in center (15 cm) zone in open field test. WT, n = 5 mice; α3ΔN Tg, n = 9 mice. Unpaired t-test, p = 0.5440. (C) Time spent in closed and open arm in elevated plus maze test. WT, n = 5 mice; α3ΔN Tg, n = 9 mice. Two-way ANOVA, effect of genotype, F1, 12 = 0.1428, p = 0.7122. (D-E) Mice were trained with three tone (2.8 kHz, 85 dB, 30 s)-shock (0.5 mA, 2 s co-terminated with the tone) pairings in fear conditioning apparatus. (D) Contextual fear memory was tested in the same training apparatus for 5 min at 24 h after the training. WT, n = 11 mice; α3ΔN Tg, n = 11 mice. Unpaired t-test, p = 0.4354. (E) Auditory fear memory was tested in another chamber with the same tone for 3 min at 48 h after the training. WT, n = 11 mice; α3ΔN Tg, n = 11 mice. Unpaired t-test, p = 0.2260. Only male mice were used for open field and elevated plus maze, whereas both male and female mice were used for fear conditioning tests. Data are mean ± SEM. [file 13041_2020_548_MOESM1_ESM.pdf]
